# Biological mortality bias in diaphyseal growth of contemporary children: Implications for paleoauxology

**DOI:** 10.1002/ajpa.24486

**Published:** 2022-01-29

**Authors:** Laure Spake, Robert D. Hoppa, Soren Blau, Hugo F. V. Cardoso

**Affiliations:** ^1^ Religion Programme and Centre for Research on Evolution, Belief and Behaviour University of Otago Dunedin New Zealand; ^2^ Department of Anthropology Western Washington University, Bellingham Washington USA; ^3^ Department of Anthropology University of Manitoba Winnipeg Canada; ^4^ Forensic Pathology The Victorian Institute of Forensic Medicine Southbank Victoria Australia; ^5^ Department of Forensic Medicine Monash University Melbourne Victoria Australia; ^6^ Department of Archaeology and Centre for Forensic Research Simon Fraser University Burnaby Canada

**Keywords:** bioarchaeology, growth and development, long bone, osteological paradox, skeletal growth profiles

## Abstract

**Objectives:**

Biological mortality bias is the idea that individuals who comprise skeletal samples (non‐survivors) are a specific subset of the overall population, who may have been exposed to greater stress during life. Because of this, it is possible that studying growth in a skeletal population misrepresents the growth and health of survivors in that population. Using a modern sample, this study investigates whether biological mortality bias in growth may be present in archaeological skeletal samples.

**Materials and methods:**

Postmortem computed tomography scans of 206 children aged under 13 years were collected from two institutions in the United States and Australia. The sample was separated into children who died from natural causes as proxies for non‐survivors and from accidental causes as proxies for survivors. Differences in long bone length for age were assessed through analysis of covariance (ANCOVA) and z‐score analysis, and these results were compared with studies linking anthropometrics and mortality risk in nonindustrialized societies.

**Results:**

Differences in growth favoring survivors were greater for girls than for boys and seemed to increase over age. The effect in nonindustrialized societies was 1.5 to 5 times the magnitude of that in our contemporary sample.

**Conclusions:**

A greater growth delay in girls than in boys has been documented in historical identified collections, and skeletal samples consistently become more stunted relative to modern standards over the course of growth. Our findings on biological mortality bias could explain part of these growth delays and impact interpretations of past ontogenetic environments.

## 1 INTRODUCTION

In *The Osteological Paradox*, Wood et al. (1992) outline a series of scenarios in which bioarchaeological conclusions drawn from skeletal samples might not reflect the health of the surviving segment of the population. Shortly after, Saunders and Hoppa (1993, p. 129) coined the term *biological mortality bias*, referring to the “physiological and morphological difference between those who die and those who survive.” Biological mortality bias is a problem for all bioarchaeological analysis, but it is especially problematic for paleoauxology, or the study of growth in the past (Tillier, 1995), because growth is known to be environmentally sensitive (Bogin, 1999). This property has made growth a popular indicator of past stress environments (Cardoso et al., 2019; Dhavale et al., 2017; Gooderham et al., 2019; Gowland et al., 2018; Newman et al., 2019), but it also makes juvenile remains in skeletal populations highly susceptible to the effects of biological mortality bias. In this paper, we focus our attention on biological mortality bias in one aspect of skeletal growth and development, namely linear growth.

Although nearly 30 years have elapsed since *The Osteological Paradox*, relatively few studies have directly addressed its most salient findings (for reviews, see Wright & Yoder, 2003 and DeWitte & Stojanowski, 2015). Even fewer have attempted to quantify biological mortality bias in linear growth by comparing surviving and non‐surviving individuals. Saunders and Hoppa (1993) used data from published anthropometric studies of nonindustrialized populations to estimate the difference in long bone length between survivors and non‐survivors from height data. Their analysis suggested a small difference in estimated long bone length, which the authors argued would be inconsequential relative to the error inherent in age estimation. Spake and Cardoso (2019) compared cadaver lengths of contemporary children deceased from accidental (proxies for survivors) and natural (proxies for non‐survivors) causes, finding small but consistent differences between the groups. This study also used a sample of girls admitted to a tuberculosis sanitarium to examine the relationship between anthropometrics and survivorship in a deceased sample, finding that weight was a better predictor of survivorship than height.

One further study on biological mortality bias in growth (Holland, 2013) focused on whether interpretations of past population health changed when results were based on the skeletal remains of children (non‐survivors) or adults (survivors). Holland matched juveniles and adults by birth year, considering adults to be survivors relative to the juveniles who were non‐survivors of childhood. Holland (2013) found no difference in the interpretation of growth when based on the non‐survivors as opposed to the survivors. This study included very few individuals younger than 15 years at death, but importantly the individuals under 15 years at death showed a tendency to be smaller for age than the non‐survivor individuals aged more than 15 years at death. This approach offers a promising framework for studying biological mortality bias, but it remains to be extended to younger children.

More recently, Spake et al. (2020) drew data from a sample of full‐body postmortem computed tomography (CT) scans of contemporary children from the United States of America (USA) and Australia. This study adopted a similar approach to Spake and Cardoso's (2019), comparing long bone length for age between survivors (children deceased from accidental causes) and non‐survivors (children deceased from natural causes). Significant differences in growth between these groups were found. Subsequently, Stull et al. (2021) used CT scans of individuals 12 years of age and under from the United States and South Africa to compare dental development and long bone growth between deceased and living children. While the authors found some differences between these groups under the age of 2 years, they argued that differential growth between the groups was not consistently present across ontogeny.

While relatively little work exists in the bioarchaeological literature on this subject, a great deal of time has been dedicated to predicting survivorship using anthropometrics in the medical and health sciences literature. Due to an interest in identifying children at higher risk of death, most of these studies present mortality rates, risk ratios, or odds ratios of death at certain anthropometric cutoffs rather than true comparisons of the anthropometrics of survivors and non‐survivors (e.g., Alam et al., 1989; Bairagi & Chowdhury, 1994; Briend et al., 1986; Chen et al., 1980; Fawzi et al., 1997; Heywood, 1983; Katz et al., 1989; Lindskog et al., 1988; Olofin et al., 2013; Pelletier, 1994; Smedman et al., 1987; Vella et al., 1993; Yambi et al., 1991). These studies can be thought of as indirect evidence of mortality bias in growth because they establish that non‐survivors are smaller for age than survivors, without stating the magnitude of this difference. Some studies, however, provide direct evidence on mortality bias by comparing anthropometrics between survivors and non‐survivors (Alam et al., 1989; Bairagi et al., 1985; Billewicz & McGregor, 1982; Briend et al., 1986; Van Lerberghe, 1988; Yambi et al., 1991). Overwhelmingly, these studies found small but statistically significant differences in height for age between survivors and non‐survivors. While one study found no difference (Van Lerberghe, 1988), the remainder found that non‐survivors were shorter for age than survivors, and this difference ranged between 2% and 5% of the reference median (Alam et al., 1989; Bairagi et al., 1985; Billewicz & McGregor, 1982; Briend et al., 1986; Yambi et al., 1991). This is roughly equivalent to between one half and one full *SD* or the same in z‐score units.

The existing literature provides a starting point for evaluating mortality bias in growth but suffers from several methodological limitations. Most importantly, nearly all of the studies detailed above, both in the bioarchaeological and medical/health sciences fields, use full‐body anthropometrics as a measure of linear growth. Spake et al. (2020) and Stull et al. (2021) provided the first studies comparing long bone length, the measure of linear growth actually used by bioarchaeologists, between survivors and non‐survivors.

In this study, we offer a comparison of long bone length for age between proxies for survivors and non‐survivors as a way of quantifying biological mortality bias in archaeological skeletal samples. We do this using a sample of CT scans of contemporary deceased children taken at autopsy in the US and Australia. Children dying of certain natural causes are considered proxies for non‐survivors, and those dying of accidental causes are considered proxies for survivors as these individuals' deaths were independent of their biological status. This framework (comparing accidental and natural deaths as proxies for survivors and non‐survivors) was previously used to study differences in dental development by Cardoso and colleagues (2010) and Spake et al. (2021) and in linear growth attainment by Spake and Cardoso (2019) and by Spake et al. (2020).

## 2 MATERIALS AND METHODS

Full body postmortem computed tomography scans of children aged 12 years and under at death were obtained from two sources: the Office of the Medical Investigator (OMI), New Mexico, USA, and the Victorian Institute of Forensic Medicine (VIFM), Victoria, Australia. In both settings, scan reconstruction settings were 1.0 mm slice thickness with 0.5 slice spacing or better. Within each source, deaths occurring from both accidents and natural deaths were targeted and taken to represent survivors and non‐survivors respectively.

In order to best replicate an archaeological skeletal sample, the natural death sample was carefully selected. With the onset of industrialization, roughly beginning as early as the 1750s in Europe and as late as the 1940s in parts of the world such as Africa and Southeast Asia, causes of child deaths have changed profoundly (Omran, 1971). Advances in modern medicine mean that children suffering from serious illnesses which persisted after the epidemiological transition can survive much longer than they would have in the past. The effects of these factors on growth status is not known, and it is conceivable that these could either improve or worsen growth status in non‐survivors. To reduce these confounding variables, a brief review of historical and anthropological demographic studies of pre‐epidemiological transition groups was conducted (e.g., Birn et al., 2010; Chandrasekhar, 1959; Dyson, 1997; Early & Peters, 2000; Gurven et al., 2007; Hill & Hurtado, 1996; Howell, 1979; Layrisse et al., 1977; McGregor et al., 1961; Patterson, 1979; Selya, 2004; Sundin, 1995; Wyon & Gordon, 1971). This review was augmented by theoretical and review articles on epidemiological transitions (e.g., Omran, 1971; Santosa et al., 2014; Zuckerman et al., 2014). These reviews produced selection criteria that excluded the following causes of death or contributing factors from the sample: cancers of all types; congenital malformations where the death occurred after 1 month of life except where the condition is known to be asymptomatic; complications of extreme prematurity; other causes of death when the child was also noted to have been born severely prematurely; fetal deaths; serious genetic diseases where they would require medical support to sustain life; chronic disease where documentation indicated that the child was hospitalized multiple times or for extended periods of time; and any other disease or pathology that would have necessitated hospitalization to ensure survival.

**FIGURE 1:**
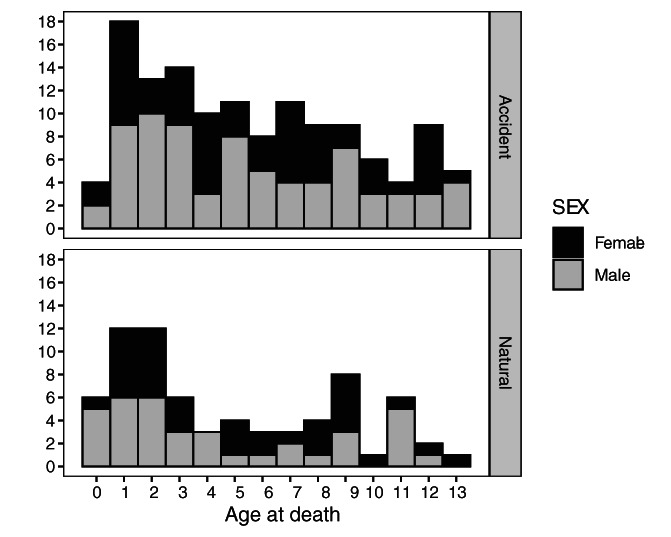
Age distribution of the total sample by manner of death and sex

The final sample was composed of 201 individuals (131 accidental and 70 natural deaths). Of these, 94 were from the OMI (70 accidental and 24 natural deaths) and 107 were from VIFM (61 accidental and 46 natural deaths). A breakdown of the sample by age, sex, and manner of death is available in Figure 1, and a more detailed breakdown of the sample by country of origin is available as Table S1. The age distribution of the sample reflects historic and contemporary mortality distributions across childhood (Chamberlain, 2006; Lewis, 2007, 2018), and so it includes more young individuals (i.e., under the age of 5 years) than it does older individuals. Natural deaths were primarily from infectious diseases (53%) but also included noninfectious (36%) causes (see Table 1). Infectious causes of death included respiratory infections (pneumonia and bronchitis), central nervous infections (e.g., meningitis, nonspecified inflammation or infection of the brain or spinal cord), and other infectious causes of death (e.g., multiple infections, sepsis, streptococcal infections, myocarditis). Noninfectious causes of death included congenital malformations (all idiopathic malformations of the heart), respiratory diseases (all asthma), and other causes (e.g., anaphylactic shock, dehydration, unsafe sleeping environments, appendicitis, intussusception, and ischemia of the bowels). There were also a substantial proportion of deaths listed as undetermined causes (*n* = 8 or 11% of the cases). These were included in the sample because they attributed to unknown causes but to a natural, and not unknown, manner of death.

**TABLE 1:** Number (N) and percentage (%) of causes of death included in the natural death sample (total number of individuals = 71)

Cause of death	*N*	%
**All causes**	71	100
**Infectious causes**	37	52
Respiratory infections (pneumonia and bronchitis)	19	27
Central nervous system infections (e.g., meningitis, nonspecified inflammation or infection of the brain or spinal cord)	6	8
Other infectious causes (e.g., multiple infections, sepsis, streptococcal infections, myocarditis)	12	17
**Noninfectious causes**	26	37
Congenital malformations (idiopathic malformations of the heart)	3	4
Respiratory disease (asthma)	6	9
Other non‐infectious causes (e.g., anaphylactic shock, dehydration, unsafe sleeping environments, appendicitis, intussusception, and ischemia of the bowels)	17	24
**Undetermined**	8	11

**FIGURE 2:**
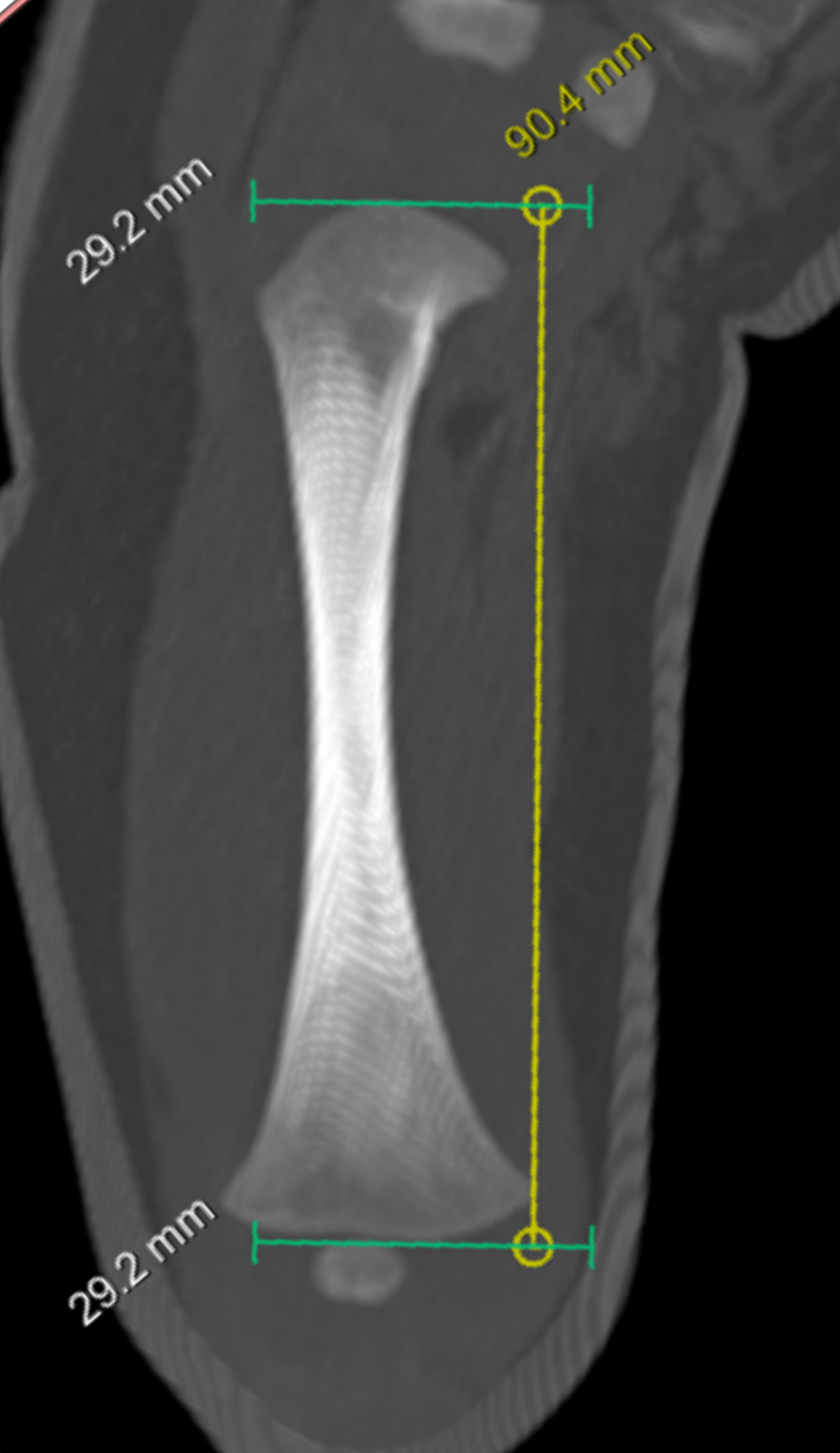
Example of the measurement of a long bone diaphysis using thin slab maximum intensity projection (slab MIP) visualization. The most proximal and distal points of the bone are simultaneously visualized although they occur at different places in the *z*‐axis of the image

For each included individual, maximum diaphyseal lengths were collected from each of the long bones: humerus, radius, ulna, femur, tibia, and fibula. Lengths were measured directly using the digital imaging and communications (DICOM) viewer. Two viewers were used: Syngo.via for the VIFM scans, a proprietary DICOM viewer from Siemens Healthineers; and Dragonfly 3.6 for Windows for the OMI scans, used under a noncommercial license, Object Research Systems (ORS) Inc., software available at http://www.theobjects.com/dragonfly. The protocol for measurement of the long bones uses a thin slab maximum intensity projection (slab MIP) aligned to replicate the measurement plane of an osteometric board (Spake et al., 2020). Slab MIP visualization condenses the information contained in a series of contiguous slices known as a slab by displaying only the value of the densest voxel along the depth of the slab (Dalrymple et al., 2005; Furlow, 2014). This essentially collapses the three‐dimensional structure of the bone into two dimensions (Figure 2). Using slab MIP ensures that the proximal and distal maximae of the bones can be simultaneously visualized on the measurement plane, yielding high measurement replicability and accuracy (Spake et al., 2020).

**FIGURE 3:**
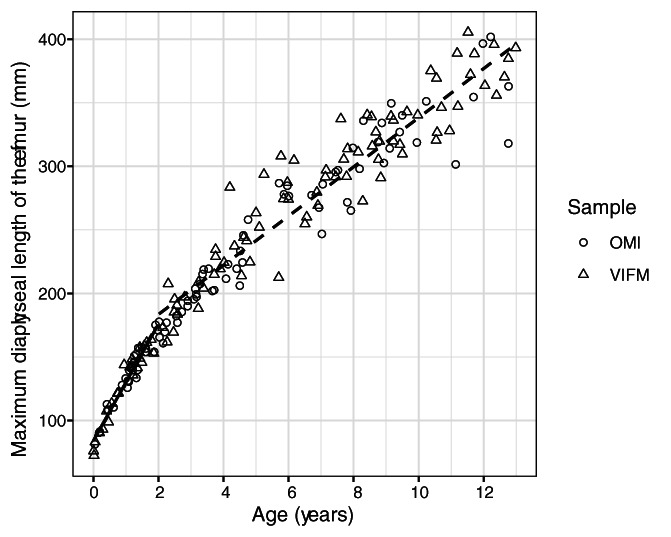
Illustration of the nonlinear relationship between age and maximum diaphyseal length of the femur. Two linear regressions demonstrate that the splitting of the sample at age two yields roughly linear relationships between the independent and dependent variables

In order to assess whether the OMI and VIFM samples could be combined for further analysis, the samples were compared with an analysis of covariance (ANCOVA) taking into consideration age, sample, sex, and the interaction between the two. The ANCOVA analysis was conducted for the accidental and natural death groups separately to ensure that the samples were similar within each test group. ANCOVA analysis assumes a linear relationship between the predictor and response variables. However, growth velocity is not uniform throughout ontogeny: it is the highest in the first 2 years of life, after which growth velocity slows until the adolescent growth spurt (Humphrey, 2003). Thus, ANCOVA analysis was performed separately for individuals younger than 2 years at death and individuals 2 years and older at death. This allowed for two linear relationships to be modeled (Figure 3). ANCOVA assumptions were tested using Levene's tests for homogeneity of variances and by building a second model with interactions between the grouping variables and covariate (age) for equality of slopes.

Once the samples were pooled, a second set of ANCOVA analyses was conducted to explore the effect of manner of death (accidental versus natural deaths) on bone length for age. Separate analyses were conducted for children under and over 2 years of age. Boys and girls are known to vary in mortality profiles, particularly at the beginning of adolescence when risk‐seeking behavior increases (Institute of Medicine, 2003; Sorenson, 2011). Thus, manner of death, sex, and the interaction between these factors were included as predictors in the analysis. ANCOVA assumptions were again tested using Levene's tests for homogeneity of variances and building a second model with interactions between the grouping variables and covariate (age) for equality of slopes.

While ANCOVA analysis allows the comparison of raw values of long bone length between the manner of death groups, the separation of the sample at 2 years of age hampers comparison of results across the entire age range. In order to compare results between children of all ages, *z*‐score analysis was also conducted. *Z*‐scores are the preferred tool for comparing growth between groups of children because they quantify deviation from an expected measurement for age (e.g., height, length, weight), and thus enable comparison of growth across children of different ages. For more discussion on the advantages of *z*‐scores in bioarchaeological growth studies, see Spake and Cardoso (2021) and for the use of *z*‐scores in population health see WHO Expert Committee on Physical Status (1995). *Z*‐scores for bone length for age were calculated from an interpolation of the Maresh (1943, 1955, 1970) reference data (Spake & Cardoso, 2021). This interpolation gives sex‐specific and sex‐combined reference values at 1 month intervals from birth to 12 years, removing the error due to rounding age down to the last attained threshold, sometimes by as much as 6 months. The interpolated values allowed *z*‐scores to be calculated for all children aged birth through 144 months (0–12 years) at death.

Z‐scores were compared between the manner of death groups using Welch's *t* tests supplemented by Cohen's *d* for effect size as suggested by Smith (2018). Welch's *t* tests are recommended as the default alternative over Student's *t* test as Welch's *t* test is known to be more robust when the homogeneity of variances assumption is violated, without losing much robustness when the assumption is not violated (Delacre et al., 2017). We provide Cohen's *d* as an effect size statistic to complement the *p*‐value, which is heavily affected by sample size (i.e., a large effect studied with a small sample may yield high *p*‐values, or a small effect studied with a very large sample can yield low *p*‐values). Effect size is a quantification of the magnitude of the difference in *z*‐scores between the two groups, relative to the variance within the groups. Effect sizes give the scientific reader an idea of potential biological importance of the effect without reference to sample size.

*t* tests were run for the entire age range, and then repeated for age groups as follows: infant (0–2.99 years); child (3–6.99 years); juvenile (7–12.99 years). These age groups were adapted from the life history stages as proposed by Bogin (1999) that define the beginning of adolescence at 10 years for females and 12 years for males. Due to the truncation of the sample at the beginning of the 12th year of life, an adolescent category would have included few individuals and been composed predominantly of females. Thus, we opted to collapse the adolescent life history stage into the juvenile stage. Analysis by age group was conducted for two reasons. First, cause of death profiles change across childhood as children become more independent from their caregivers and begin to engage in adult behaviors (Bogin, 1999; Institute of Medicine, 2003). Second, archaeological studies of growth consistently document greater delay in linear growth with an increase in age (e.g., Cardoso, 2005; Cardoso et al., 2019; Dori et al., 2020; Ives & Humphrey, 2017). This is congruent with the increase in variation in growth status that occurs over the growth period (Bogin, 1999). Because of this increase in variation, and the compounding effects of environmental insults on growth over time, there is reasonable suspicion that biological mortality bias in growth may be differently expressed in younger versus older individuals.

In order for comparisons between the accidental and natural death groups to be representative of the biological mortality bias found between survivors and non‐survivors, accidental death victims should represent a true cross‐section of the healthy and surviving children in the population. To test this, the cadaver lengths of the accidental death victims were compared with the CDC‐2000 growth reference (Kuczmarski et al., 2000) using *z*‐scores. Fatal accidents are known to disproportionately affect children from lower socioeconomic status groups (Cubbin & Smith, 2002; Institute of Medicine, 2003; Laflamme et al., 2010; Singh & Kogan, 2007). In turn, children from lower socioeconomic status groups are known to be growth delayed relative to their wealthier peers, even in developed nations (Crooks, 1999; Ehouxou et al., 2009; Grimberg et al., 2009; Moffat et al., 2005; Moffat & Galloway, 2007). Thus, there was reason to believe that the accidental death victims may be small for age compared with the healthy population of children. The CDC‐2000 reference was selected because it provides the best description of growth in US children and can reasonably be expected to fit Australian children as well. Although newer references have been published by the World Health Organization (WHO), these references only apply to children under 5 years (WHO‐2006 [WHO Multicenter Growth Reference Study Group, 2006]), or consist of data from US children growing up in the early to middle 20th century (WHO‐2007 [de Onis et al., 2007]). Cadaver length was available for 120 of the 131 accidental death victims. For children under 2 years at death, cadaver length was compared with recumbent length references. For children 2 years and over at death, cadaver length was first converted to stature using a regression equation (Krishan & Sharma, 2002). *Z*‐scores were then calculated relative to the stature reference parameters. *Z*‐scores less than −6 and greater than 6 were considered outliers as suggested by the WHO (WHO Multicenter Growth Reference Study Group, 2006) and removed from analysis. The *z*‐scores were then compared with the reference mean, or zero, using a one‐sample *t* test paired with the effect size statistic (Cohen's *d*).

Lastly, to gain a sense for how this sample compared with less industrialized populations, results from this analysis were compared with those of a series of papers that compared anthropometrics of survivors and non‐survivors in nonindustrialized populations (Alam et al., 1989; Bairagi et al., 1985; Briend et al., 1986; Yambi et al., 1991). These studies were part of the larger group of studies tying anthropometrics to survivorship (see Introduction for more details on this group of studies) and were selected because the means and standard deviations for the anthropometrics for survivors and non‐survivors could be gleaned from the papers. These studies were primarily conducted in the 1970s–1980s, in rural Matlab (Alam et al., 1989; Bairagi et al., 1985) and urban Dhaka in Bangladesh (Briend et al., 1986); and the rural Iringa region of Tanzania (Yambi et al., 1991). Because these studies focused on individuals under 5 years of age, we restricted this analysis to the same age range. The recumbent lengths or calculated statures (see above) were compared with the NCHS‐1977 growth reference (Hamill et al., 1977) by calculating the percentage of the age‐specific median attained by the child. Percentage of the median scores and the NCHS‐1977 reference were used to match the methods used by the comparative studies. Percent of the median scores are similar to *z*‐scores in that both express deviation from the reference mean/median and can range above and below that mean/median, but are different in that percent of the median scores are not standardized for age‐specific variation. Once percent of the median height score was calculated for each individual, scores were compared across manner of death groups using *t* tests and Cohen's *d* for effect size.

## 3 RESULTS

**FIGURE 4:**
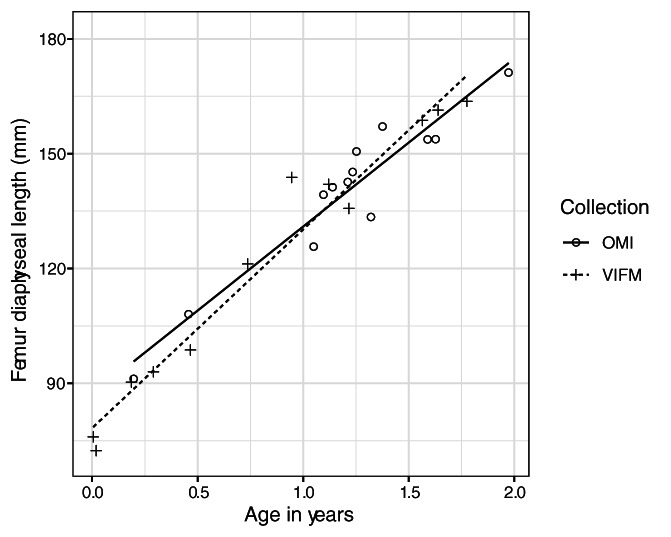
Femur diaphyseal length for age for children under 2 years of age, for each sample separately

When comparing the accidental and the natural death groups across the samples to evaluate if it was appropriate to pool them, ANCOVA analysis revealed no consistent difference in bone length for age across the OMI and VIFM samples (Table 2 and 3). In the under 2 years age group of the natural deaths, Levene's tests found differing variances across the samples for every bone. Plotting suggests that this heterogeneity in variance could be due to uneven sampling across the age range when comparing institutions: for example in the youngest ages, there are more individuals from the VIFM than OMI sample (Figure 4). Turning to the over 2 years age group, the only significant difference between the samples was in femur length for children in the accidental death group. It is possible that this difference represents a statistical false‐positive, as comparisons for the other five bones showed no statistical significance (*p*‐values ranging from 0.21 to 0.53). For this reason, the samples were considered to be sufficiently similar in their long bone growth and were pooled for further analysis. While the Levene's tests showed significant heterogeneity in variance across the two samples, this test merely indicates that if one wants to increase confidence that a statistically significant result of the ANCOVA test indeed reflects a rejection of the null hypothesis (which is that the two samples are not different), one should use a parametric test. Since no consistent differences in long bone length for age between the samples were found, nonparametric tests were not used. The sex variable introduced into the ANCOVA showed no significant effect in those under 2 years at death (Table 2). However, in the over 2 years at death group, the sexes differed for every bone in the natural death group and for the radius in the accidental death group (Table 3). While these differences did not affect the decision to pool the samples, the sexes were considered to potentially differ in their expression of mortality bias, and sex was kept as a variable of interest in the following analyses.

**TABLE 2:** Results of the ANCOVA analysis to pool the two samples, for the natural deaths

	Age < 2 years					Age ≥ 2 years				
Bone	*N* OMI	*N* VIFM	Effect	F	*p*	*N* OMI	*N* VIFM	Effect	F	*p*
Humerus	13	12	Sample^a^	0.23	0.64	11	30	Sample	1.76	0.19
			Sex	0.91	0.35			Sex	7.78	0.01
			Int.	1.81	0.19			Int.	0.28	0.60
Radius	13	12	Sample^a^	0.07	0.79	11	32	Sample	1.16	0.29
			Sex	0.71	0.41			Sex	17.97	<0.01
			Int.	1.56	0.23			Int.	0.76	0.39
Ulna	12	12	Sample^a^	0.24	0.63	11	31	Sample	2.15	0.15
			Sex	0.43	0.52			Sex	11.96	<0.01
			Int.	1.35	0.26			Int.	1.16	0.29
Femur	13	12	Sample^a^	0.01	0.93	13	12	Sample	2.15	0.15
			Sex	1.07	0.31			Sex	6.95	0.01
			Int.	0.05	0.83			Int.	0.01	0.93
Tibia	13	12	Sample^a^	0.00	0.97	11	31	Sample	0.23	0.64
			Sex	1.71	0.21			Sex	7.93	0.01
			Int.	0.04	0.84			Int.	0.19	0.67
Fibula	13	12	Sample^a^	0.03	0.86	11	33	Sample	1.39	0.25
			Sex	1.09	0.31			Sex	7.27	0.01
			Int.	0.19	0.67			Int.	0.39	0.54

*Note*: Number of individuals from the OMI (*N* OMI), from VIFM (*N* VIFM), and F and *p*‐values for the effect of sample, sex, and the interaction between the two on long bone length.

^a^ Levene's test for this grouping variable reveals heterogeneity of variances (*p* < 0.05).

**TABLE 3:** Results of the ANCOVA analysis to pool the two samples, for the accidental deaths

	Age < 2 years					Age ≥ 2 years				
Bone	*N* OMI	*N* VIFM	Effect	F	*p*	*N* OMI	*N* VIFM	Effect	F	*p*
Humerus	19	10	Sample	0.02	0.89	48	42	Sample	1.53	0.22
			Sex	1.37	0.25			Sex	0.02	0.88
			Int.	3.24	0.08			Int.	2.60	0.11
Radius	19	10	Sample	0.63	0.44	50	46	Sample	0.40	0.53
			Sex	0.29	0.60			Sex	5.84	0.02
			Int.	3.63	0.07			Int.	2.78	0.10
Ulna	19	10	Sample	0.32	0.58	50	45	Sample	0.42	0.52
			Sex	1.03	0.32			Sex	2.38	0.13
			Int.	6.33	0.02			Int.	2.39	0.13
Femur	18	10	Sample	0.11	0.75	47	45	Sample	5.18	0.03
			Sex	2.84	0.11			Sex	0.05	0.82
			Int.	2.18	0.15			Int.	2.00	0.16
Tibia	19	10	Sample	0.32	0.58	48	49	Sample	1.22	0.27
			Sex	1.27	0.27			Sex	0.09	0.77
			Int.	4.03	0.06			Int.	3.05	0.08
Fibula	19	10	Sample	0.92	0.35	48	49	Sample	1.62	0.21
			Sex	1.47	0.24			Sex	0.00	0.99
			Int.	2.84	0.11			Int.	1.58	0.21

*Note*: Number of individuals from the OMI (*N* OMI), from VIFM (*N* VIFM), and F and *p*‐values for the effect of sample, sex, and the interaction between the two (Int.) on long bone length.

**FIGURE 5:**
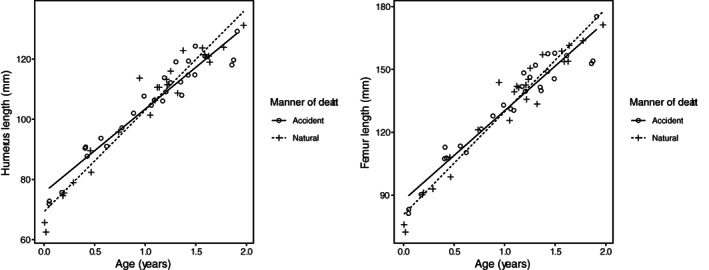
Visualization of the ANCOVA analysis comparing the manner of death groups in the under 2 years at death group. Unequal slopes between the manner of death groups were found for the humerus (left), but not for the femur (right)

When comparing the manner of death groups with an ANCOVA analysis, Levene's tests revealed no cases where homogeneity in variance was violated. In the tests, the under 2 years at death age group showed little differences in long bone length for age in either the manner of death groups or the sexes (Table 4). For the sexes combined, the groups did not differ significantly in bone length for age across any of the long bones. In all comparisons except the radius, sex showed larger values than manner of death (F values ranging from 0.01 to 1.17 for manner of death, and 0.98 to 3.54 for sex). Plotting showed that the slopes of the manner of death groups were not quite equal (Figure 5): the youngest individuals had smaller long bones for age in the natural death group relative to the accidental death group, while the magnitude of this difference was reduced or reversed in the older individuals. This was only significant for the humerus, where assumptions testing found inequality of slopes. Because sex did not have a significant impact on long bone length for age in this group, a sex‐specific ANCOVA analysis was not pursued.

**TABLE 4:** Results of the ANCOVA analysis comparing the manner of deaths, for the under 2 years at death category

Bone	*N* Accident	*N* Natural	Effect	F	*p*
Humerus	29	25	MOD^a^	0.01	0.92
			Sex	1.88	0.18
			Int.	0.00	0.98
Radius	29	25	MOD	1.17	0.28
			Sex	0.64	0.43
			Int.	0.10	0.76
Ulna	29	25	MOD	0.61	0.44
			Sex	0.98	0.33
			Int.	0.01	0.93
Femur	28	25	MOD	0.01	0.91
			Sex	3.54	0.07
			Int.	0.07	0.80
Tibia	26	25	MOD	0.02	0.89
			Sex	2.77	0.10
			Int.	0.322	0.57
Fibula	29	25	MOD	0.01	0.93
			Sex	2.02	0.16
			Int.	0.13	0.72

*Note*: Number of individuals in the accident (*N* Accident) and natural (*N* Natural) death groups, and F and *p*‐values for the effects of manner of death (MOD), sex, and the interaction between the two (Int.) on long bone length.

^a^ For this predictor, the groups showed significantly different slopes (*p* < 0.05) when the model was built with interaction between this term and age.

**FIGURE 6:**
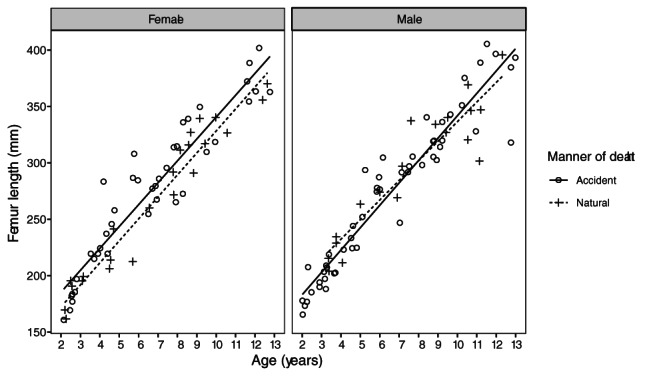
Visualization of the ANCOVA analysis comparing the manner of deaths in boys and girls separately. The graphs show a difference between the manner of death groups in the females that is not found in the males

Among individuals aged 2 years and over at death, significant differences in long bone length for age were observed for the sexes for the radius and ulna and for the interaction term for the humerus, radius, tibia, and fibula (Table 5). The significance of the interaction term across multiple comparisons suggests that sex could be confounding the relationship between long bone length and manner of death. This was confirmed visually (Figure 6). Thus, ANCOVA analysis was repeated for the sexes separately, the results of which are also reported in Table 5. For the females, F values ranged from 5.80 to 7.52 and differences were statistically significant in each comparison. Because multiple bones were compared across the same individuals, we adjusted *p*‐values for females over the age of 2 years using the false discovery rate (FDR) method (Benjamini & Hochberg, 1995) to ensure that our findings were not due to multiple comparisons. The FDR method is recommended for *p*‐value adjustment as it allows adjustment with the least inflation of type 2 error (Jafair & Ansari‐Pour, 2019). In all cases, *p*‐values remained below the 0.05 threshold after adjustment. For the males, F‐values ranged from 0.05 to 0.36 and were never statistically significant.

**TABLE 5:** Results of the ANCOVA analysis comparing the manner of deaths for the 2 years and older at death group

				Sexes combined		Females			Males	
Bone	*N* Accident	*N* Natural	Effect	F	*p*	F	*p*	Adj. *p*	F	*p*
Humerus	90	41	MOD	2.80	0.10	7.52	0.01	0.02	0.18	0.67
			Sex	1.95	0.17	‐	‐	‐	‐	‐
			Int.	4.74	0.03	‐	‐	‐	‐	‐
Radius	96	43	MOD	2.28	0.13	5.88	0.02	0.02	0.36	0.55
			Sex	19.02	<0.01	‐	‐	‐	‐	‐
			Int.	4.44	0.04	‐	‐	‐	‐	‐
Ulna	95	42	MOD	2.94	0.09	5.94	0.02	0.02	0.12	0.73
			Sex	9.91	<0.01	‐	‐	‐	‐	‐
			Int.	3.66	0.06	‐	‐	‐	‐	‐
Femur	92	42	MOD	2.78	0.10	5.80	0.02	0.02	0.05	0.83
			Sex	1.25	0.27	‐	‐	‐	‐	‐
			Int.	3.19	0.08	‐	‐	‐	‐	‐
Tibia	97	44	MOD	3.08	0.08	6.57	0.01	0.02	0.15	0.70
			Sex	3.50	0.06	‐	‐	‐	‐	‐
			Int.	4.79	0.03	‐	‐	‐	‐	‐
Fibula	97	44	MOD	2.83	0.10	6.17	0.02	0.02	0.12	0.73
			Sex	2.19	0.14	‐	‐	‐	‐	‐
			Int.	4.2	0.04	‐	‐	‐	‐	‐

*Note*: Number of individuals in the accident (*N* Accident) and natural (*N* Natural) death groups, and F and *p*‐values for the effects of manner of death (MOD), sex, and the interaction between the two (Int.) on long bone length. Analysis was performed for the sexes combined, then for the sexes separately. For females, adjusted *p*‐values (Adj. *p*) are also given and were adjusted using the false discovery rate (FDR) method.

**FIGURE 7:**
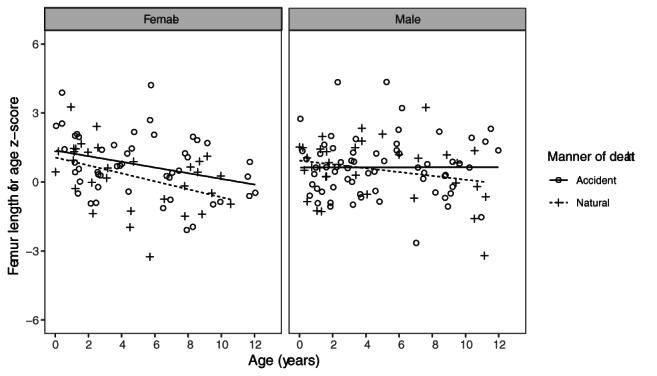
Femur length for age *z*‐score for the manner of death groups in each sex separately

For the z‐score analysis, means and standard deviations for each manner of death group as well as results for the *t* tests and Cohen's *d* for comparisons between them are reported in Table 6 for the femur as representative of the other long bones. Similar tables for the humerus, radius, ulna, tibia, and fibula are available in Tables S2 to S6 respectively. Generally speaking, the mean *z*‐scores for the accidental death group were more positive than the natural death group, indicating that the accidental death group tended to have larger bone lengths for age. The five exceptions to this were all in the infant group, in the radius, ulna, femur, tibia, and fibula, where the natural deaths had larger *z*‐scores. The largest differences between the manner of death groups were consistently found in the child age group, where effect sizes (Cohen's *d*) ranged from 0.42 to 0.61 but did not reach significance. The juvenile group showed little difference between the groups. When considering the sexes separately, the manner of death groups were significantly different for girls in the child age group, and for every bone for this group, but were not different in any comparisons for the boys (Figure 7). To ensure that the statistically significant results for the girls in the child age group were robust to the type of tests used, we compared the results of Welch's *t* tests to those obtained from Mann–Whitney U‐tests, a nonparametric alternative to the *t* test. *p*‐values for both sets of tests were adjusted using the FDR method. The results were robust to both the type of test used and to the adjustment of *p*‐values (Table S7).

**TABLE 6:** Number of individuals (*N*) and mean and *SD* for femur length *z*‐scores for each of the manner of deaths

	Accident			Natural					
	*N*	Mean	*SD*	*N*	Mean	*SD*	*t*	*p*	*d*
Sexes combined									
Infant	43	0.78	1.26	29	0.82	1.09	−0.15	0.88	0.04
Child	36	1.12	1.58	15	0.16	1.58	1.99	0.06	0.61
Juvenile	36	0.28	1.25	20	0.04	1.39	0.64	0.52	0.19
Total	115	0.73	1.40	64	0.42	1.34	1.45	0.15	0.22
Females									
Infant	19	0.95	1.29	14	1.10	1.14	−0.35	0.73	0.12
Child	16	1.36	1.84	7	−0.79	1.49	2.96	0.01	1.23
Juvenile	15	0.19	1.31	10	−0.12	0.95	0.68	0.50	0.26
Total	50	0.85	1.54	31	0.28	1.38	1.74	0.09	0.39
Males									
Infant	24	0.65	1.24	15	0.57	1.01	0.22	0.83	0.07
Child	20	0.93	1.36	8	0.99	1.17	−0.12	0.91	0.05
Juvenile	21	0.35	1.24	10	0.20	1.78	0.24	0.81	0.11
Total	65	0.64	1.28	33	0.56	1.31	0.29	0.77	0.06

*Note*: The *t* and *p* values for *t* tests between them and Cohen's *d* for effect size are given. Values are calculated for the age groups separately and for the total sample.

**FIGURE 8:**
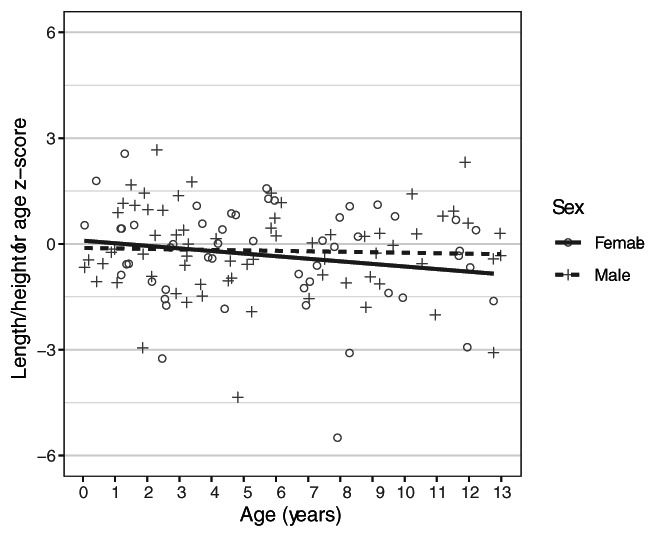
*Z*‐score over age for cadaver length and stature for the accidental death victims when compared with the CDC‐2000 growth reference

Comparison to the CDC‐2000 growth reference showed that the accidental death group was shorter for age than the reference, as exemplified by negative mean *z*‐scores (Table 7). For the sexes combined, individuals were increasingly short for age with increasing age. The difference was significant in the juvenile age group (7–12.99 years) and overall sample. The sexes differ in their *z*‐score patterning. Females followed the pattern of decreasing average z‐score over age, except in the child age group, where no real difference was found between the manner of deaths (as evidenced by a very small Cohen's *d*). Males showed less of a trend toward increasing growth deficit with age than females did (Figure 8). Effect size for the girls reached 0.42 while only reaching 0.31 for the boys, reinforcing that girls deviated from the reference more than boys did.

**TABLE 7:** Comparison of survivor cadaver lengths to the CDC‐2000 reference

	*N*	Mean	*SD*	*t*	*p*	*d*
Sexes combined						
Infant	38	−0.05	1.33	−0.23	0.82	0.04
Child	37	−0.20	1.25	−0.95	0.35	0.16
Juvenile	45	−0.47	1.39	−2.25	0.03	0.36
Total	120	−0.25	1.33	−2.07	0.04	0.19
Females						
Infant	16	−0.30	1.40	−0.85	0.49	0.21
Child	16	0.09	1.08	0.34	0.74	0.08
Juvenile	20	−0.70	1.65	−1.88	0.07	0.42
Total	52	−0.33	1.43	−1.67	0.10	0.23
Male						
Infant	22	0.13	1.27	0.49	0.63	0.10
Child	21	−0.41	1.34	−1.41	0.17	0.31
Juvenile	25	−0.29	1.15	−1.24	0.23	0.25
Total	68	−0.19	1.25	−1.25	0.22	0.15

*Note*: Number of individuals (*N*), mean, and *SD* are given for each group, and the *t* and *p*‐values, and Cohen's *d* for effect size are given for the one sample *t* test.

When comparing the mortality effect found in this analysis to those found in anthropometric studies of nonindustrialized groups, this analysis showed the smallest difference (Table 8). The difference between the manner of deaths in the percent of the median scores for length/stature in this analysis was 1.03 (*t* = 0.70, *p* = 0.47), while in other studies it ranged from 1.60 to 4.95. The *t*‐values available for two of these studies ranged from 3.00 to 5.34 (*p* < 0.05), and were available for the studies showing the smallest and second‐largest differences between survivors and non‐survivors.

**TABLE 8:** Number of individuals (*N*) and mean and *SD* for the NCHS percentage of median scores for children under 5 years of age in each of the manner of death groups

	Accident/survivors			Natural/non‐survivors						
	*N*	Mean	*SD*	*N*	Mean	*SD*	x1–x2	*t*	*p*	*d*
This study	60	98.93	5.56	24	97.90	6.29	1.03	0.70	0.47	0.15
Bairagi et al. (1985)	919	85.69	5.78	19	80.74	3.96	4.95	5.34	<0.01	‐
Briend et al. (1986)	318	76.00	8.00	34	71.00	9.00	5.00	‐	<0.05	‐
Alam et al. (1989)	9801	89.00	4.20	60	85.70	5.70	3.30	‐	‐	‐
Yambi et al. (1991)	2364	92.30	4.10	88	90.70	4.90	1.60	3.00	<0.01	‐

*Note*: The difference between the means (x1–x2), *t* and *p*‐values for *t* test between them, and Cohen's *d* for effect size are also given. The same information is given for the comparative studies (*t* and *p*‐values) where they were available from the studies.

## 4 DISCUSSION

In children under 2 years of age, analysis revealed no clear difference between survivors and non‐survivors. However, rates of increase in bone length varied between survivors and non‐survivors, as evidenced by inequality of slopes across the manners of death in at least one bone, the humerus. Plotting suggested that for the humerus, the youngest individuals (less than 0.5 years or 6 months) were particularly small for age. *Z*‐score analysis was helpful for this age group because it allowed comparison of survivors and non‐survivors in a way that was free from the assumptions of ANCOVA analysis. The *z*‐score analysis confirmed that there was no discernable biological mortality bias effect in the infant (0–2.99 years) age group as a whole.

Importantly, the two youngest non‐survivors were considerably smaller for age than survivors in the first 2 months of life. The *z*‐scores for all of the bones of these two non‐survivors ranged from −2.17 to −0.77 and from −3.85 to −1.29. This is considerably smaller for age than the next smallest individual in the under 6 months age group, who is a survivor and whose *z*‐scores range from −0.56 to +0.70. Because these individuals did not live long after birth, their causes of death were revisited to screen for prematurity. Of the two non‐survivors, the smallest was specifically noted to have been a full‐term birth. There was no further information on the gestational age of the other individual, and causes of death for these individuals did not raise concern that they should have been excluded from the sample. There are two possible explanations for especially large differences in growth between survivors and non‐survivors in the youngest individuals. On one hand, this could be due to factors impacting health and growth in the gestational periods, as maternal factors are known to be a major driver of mortality in the first month of life (Abdullah et al., 2016; Battin et al., 2007; Bourgeois, 1951). However, in part because the study design excluded deaths due to severe prematurity or gestational deaths, those where cause of death was unknown, and sudden deaths in infancy, sample size is small in this age group, particularly in the accidental death group. Thus, it is possible that this difference reflects the size of the sample rather than capturing a true biological effect.

When testing for the effects of biological mortality bias in children after infancy, ANCOVA analysis revealed a sex‐based difference. Male survivors and non‐survivors never differed in long bone length for age, whereas female survivors were significantly larger than non‐survivors for all bones. *Z*‐score analysis suggested that the biological mortality bias effect was especially important for the child age category (3–7 years), and was less important for the juvenile age group (7–12 years). However, comparing survivors to the CDC‐2000 reference suggested that the survivors were also short for age compared with healthy US children, particularly in the juvenile age group. Again, this effect was larger for girls than for boys. Thus, both ANCOVA and *z*‐score analysis suggest that there exists a biological mortality bias effect in girls over the age of 3. The effect may exist in boys but to a much smaller, if any, degree.

There are several possible causes for the difference in effect between boys and girls. One potential interpretation is differential eco‐sensitivity between the sexes, whereby the growth of girls is proposed to be more buffered to the effects of environment factors as compared with that of boys (Stini, 1969; Stinson, 1985). However, this is not likely to be the cause of the difference observed here, where girls show a greater difference in growth than boys do. Another explanation is that the greater effect of mortality bias observed in girls could be due to cultural preference for males. Ives and Humphrey (2017) have shown a greater delay in skeletal growth in girls than in boys in the Bethnal Green identified skeletal sample. The delay was not observed in the Spitalfields identified sample (Humphrey, 1998), perhaps due to the higher socioeconomic status of the Spitalfields sample. Indeed, some research has suggested that differences in growth between the sexes are more pronounced in lower socioeconomic status groups (Rousham, 1999). Other studies of non‐English identified skeletal collections have shown some delay in girls, although the magnitude of the delay was inconsistent (Cardoso, 2005; Facchini & Veschi, 2004). It is unclear whether or how cultural preference could be expressed in this sample; however as previously argued, this sample is likely to reflect somewhat lower socioeconomic status. Lastly, it is possible that this difference represents some type of sampling bias, specifically that the natural death sample captures a different group of males and females. To explore whether this could be explained by cause of death, frequencies of broad and finer groups of natural causes of death (as defined in Table 1) were compared between males and females using a Fisher's exact test. There were no differences between the sexes in either the broad categories (*p* = 1.00) or in the finer categories (*p* = 0.71). However, this does not mean that there were no differences in disease experience between males and females. For example, there were more instances of respiratory infections for males, while females had more cases of myocarditis. Unfortunately, medical history beyond cause of death and certain contributing factors was not available for the children. Although we cannot investigate differential disease experiences beyond this, it is likely that the difference in biological mortality bias between boys and girls found in this study is due to differential capture of individuals by natural death processes rather than differences due to sex‐based variation in eco‐sensitivity.

In addition to providing support for ANCOVA analysis as well as comparing the magnitude of biological mortality bias over the growth period, *z*‐scores allow different body segments to be compared. This is important because different segments are known to be variably sensitive to environmental influences (Cardoso, 2005; Pomeroy et al., 2012). However, in this analysis *z*‐scores were not consistently patterned across the long bones. In the overall sample, the leg seemed to show larger difference (larger *t*‐values) between the survivors and non‐survivors relative to that of the arm, but this pattern did not hold when the sexes were considered separately. Similarly, in the overall and female samples, the proximal segments of the limbs showed larger differences between survivors and non‐survivors, but this pattern was reversed in the males. Typically, research showing differential sensitivity to environmental factors suggests that the leg is more influenced than the arm, and that the distal segments of each limb are more influenced than the proximal segments (Bogin & Varela‐Silva, 2009; Pomeroy et al., 2012). The results of this analysis do not align with these hypotheses. Greater sensitivity in the above segments should lead to higher differences between survivors and non‐survivors in the segments, and while we find some evidence that the leg is more affected than the arm, we do not find evidence that the distal segments were more affected than the proximal segments. More generally speaking, *z*‐score analysis revealed that children were advanced in growth relative to the Maresh data at birth, but became more delayed over the course of the age range (Figure 7).

This analysis is likely to be a conservative estimate of biological mortality bias in growth for two reasons. First, comparison of the accident victims to the CDC growth reference suggested that the individuals used as proxies for survivors were small for age relative to the healthy population (Table 4). This was particularly true for older children and for girls. This is important because the biological mortality bias between survivors and non‐survivors in older girls cannot be explained by tall‐for‐age survivors. In fact, in the older girls, even the survivor sample was small for age, suggesting that the biological mortality bias found in older girls is real and significant in effect. This result was expected, as previous analysis of other groups of children from these samples also revealed that accidental death victims were short for age (Spake & Cardoso, 2018). Second, previous studies in nonindustrialized groups found differences in height between survivors and non‐survivors that were roughly 1.5 to 5 times the size of the ones found in this analysis (Table 8). This portion of the analysis was restricted to individuals under 5 years at death, and it is not known how this applies to older individuals. However, our results stem from a study of modern children, who are relatively buffered from disease and nutrition‐related growth insults stemming from disease and poor nutrition. Thus, it is reasonable to expect that our results in older children are also on the low end of the range of biological mortality bias that exists in nonindustrialized groups. As a whole, biological mortality bias was most noticeable for older individuals, particularly females, and potentially also for young individuals under 6 months of age.

The implications of this study's detection of biological mortality bias in very young infants (<6 months) and older children, particularly females, for interpretations of paleoauxological results depend on two traits of the past population understudy. The first is the population‐specific magnitude of the biological mortality bias effect between survivors and non‐survivors, which we have discussed extensively above, and the second is the number of individuals in the affected age groups present in the skeletal sample studied. In past populations, children show the highest mortality in the first and second years of life, after which mortality declines and reaches low levels around 5 years of age (Chamberlain, 2006; Ives & Humphrey, 2017; Rousham & Humphrey, 2002). Mortality in the first year of life is the highest around birth, and declines thereafter (Bourgeois, 1951; Humphrey et al., 2012). After the fifth year or life, mortality remains low until late adolescence or early adulthood (Chamberlain, 2006; Omran, 1971), as deaths related to hunting, interpersonal violence, warfare, or childbearing begin to increase.

Because very young individuals are more common than older children in archaeological skeletal assemblages, the biological mortality bias found in this study in the youngest infants (less than 6 months) has the potential to bias interpretations of past population health. Deaths in the neonatal period, or the first 28 days after birth, are known to be due to endogenous factors such as genetic illness or maternal gestational factors (Bourgeois, 1951; Herring et al., 1991; Saunders & Barrans, 1999). Thus, infants dying soon after birth may have experienced disproportionate levels of gestational growth insults, producing impaired growth as reflected in the results in this study. However, in archaeological applications, this difference may or may not be meaningful. Despite their high mortality rates relative to other age groups, fetal, and perinatal remains have been notoriously difficult to fully integrate into paleoauxological analyses (Blake, 2017; Halcrow et al., 2017; Hodson, 2021). There is some disagreement as to whether very young infants are underenumerated in skeletal samples, whether due to differential diagenesis, excavation biases, or cultural burial practices (Fisk, 2018; Fisk et al., 2019; Halcrow et al., 2017; O'Neill, 2020; Saunders & Barrans, 1999). Assigning age or even detecting if birth occurred is notoriously difficult in young infants (Blake, 2017; Saunders & Barrans, 1999). While atlas dental age estimation methods sometimes imply that age at birth can be estimated from the dentition with the precision of ±2 weeks (AlQahtani et al., 2010), regression methods using the length of developing deciduous teeth have a precision of ±2 months (Cardoso et al., 2019; Liversidge et al., 1993). Histological methods, and more recently their virtual counterparts, which make use of the neonatal lines can elucidate whether the child survived the birth process or not, and can also be used to estimate age more precisely than can be done from macroscopic or metric assessment of the developing dentition (Le Cabec et al., 2017; McFarlane et al., 2014). We have shown elsewhere that dental development, at least in the permanent dentition, is buffered from the effects of biological mortality bias (Spake et al., 2021), which was consistent with the small existing literature on the topic (Cardoso et al., 2010; Holman et al., 2004; Stull et al., 2021). If more precise dental age estimation methods for use in very young individuals can be developed and consistently used, then the effect of biological mortality bias on the interpretation of growth in this age group has the potential to be important. Until then, any growth differences caused by biological mortality bias in this age group may be too small in comparison to errors introduced by methodological factors such as underenumeration, measurement error, and age estimation error, to impact paleoauxological conclusions.

Children over 3 years at death, and particularly those over 7 years, are less represented in skeletal samples as these age groups typically have lower mortality rates than younger children (Chamberlain, 2006). However, biological mortality bias in this age group was large and statistically significant, particularly for girls. To get a sense for the magnitude of mortality bias in terms of long bone lengths, we can use the z‐score formula to convert the difference in growth attainment between survivors and non‐survivors from z‐score units to centimeters of bone growth and then to months of growth. For the female child age category, the difference in mean *z*‐score is 2.15 *z*‐score units. At the beginning of this age group, 3 years, a female child with a femur length 2.15 *z*‐score units below average would match the mean femur length for female children of about 27 months of age—a difference of 11 months, or nearly a year, of growth. At the end of the child age group, at 83 months (7 years minus 1 month), a female child delayed 2.15 *z*‐scores would match the mean femur length for a female child of about 65 months of age—a difference of 18 months, or a year and a half of growth. These are perhaps not substantial shifts for a single child, but if the distribution of long bone lengths for age for non‐survivors are shifted 2.15 *z*‐score units (or in other words, 2.15 *SD*) from the mean, this represents a substantial difference between survivors and non‐survivors of a single population. Archaeological samples are generally noted to become more delayed relative to modern norms as age increases (e.g., Cardoso et al., 2019; Dori et al., 2020; Ives & Humphrey, 2017), which tends to be attributed to cumulative effects of environmental insults over time. This study, however, suggests that at least some of this delay is due to biological mortality bias. This is especially important as paleoauxological studies of known age and sex collections have consistently demonstrated greater growth delays in girls than in boys (Cardoso, 2005; Facchini & Veschi, 2004; Ives & Humphrey, 2017).

This study has several limitations, not least of which was sample size. Sampling was limited by availability of good‐quality scans, which yielded smaller sample sizes for natural deaths and particularly for individuals over the age of four (Figure 1). Additionally, the sampling approach taken limited sample size in the first months of life, as described above. When pooling the sexes, the smallest number of individuals for any age group was 14 (for the child category in the ulna *z*‐scores and *t*‐tests). However, when the sexes are examined separately, sample sizes for the natural deaths could be as small as seven individuals. Statistically speaking, *t* tests are robust to small sample sizes. However, inadequate sampling could be either masking or overemphasizing a true biological effect. For example, this sample exhibits a large amount of variation in long bone length for age *z*‐scores when compared with those of historical populations (e.g., Cardoso, 2005). There is no clear guideline on the number of individuals needed to adequately capture the growth pattern of a population or subpopulation for anthropological purposes (Corron et al., 2018), although sample sizes of 200 per age year and sex are recommended for devising growth curves (Frongillo, 2004). Unfortunately, sample size in anthropology is often dictated by availability and small samples are often unavoidable.

Another constraint of the analysis is that the manner of death groups as we have defined them may not be perfect proxies for either the survivors or non‐survivors of past populations. In contemporary populations, accidental injury deaths disproportionately affect individuals of lower socioeconomic status (Cubbin & Smith, 2002; Laflamme et al., 2010; Singh & Kogan, 2007). Thus, the accidental death group in this study may not reflect a true cross‐section of the developmental status of the surviving population, as socioeconomic status is also known to be linked to height for age, even in developed nations (Crooks, 1999; Ehouxou et al., 2009; Grimberg et al., 2009; Moffat et al., 2005; Moffat & Galloway, 2007). This assertion was supported by the comparison of the accidental death victims to the CDC‐2000 reference, which revealed overall delay relative to the reference increasing with age, particularly in the girls (Table 7 and Figure 7). Similarly, the selection criteria for natural deaths used in this study excluded some causes that could have contributed to the archaeological samples. For example, causes of deaths where the child would have required medical support were excluded because modern medicine can prolong life of children with these illnesses, potentially leading to either better or worse growth status for age relative to the growth status they would have without medical intervention. Further, the selection criteria excluded accidental or intentionally violent causes of death. Although accidental deaths and cases of interpersonal violence certainly affected children in past populations (e.g., Chandrasekhar, 1959; Early & Peters, 2000; Hill & Hurtado, 1996; Howell, 1979), we were not able to find enough information on the ages and rates at which they occur, and we decided to omit accidental deaths from the non‐survivor sample altogether. This analysis drew distinctions between survivors and non‐survivors based on cause and manner of death only. Medical records were not available to us. The final cause of death is an arbitrary distinction to draw, as it does not necessarily reflect disease experience in life.

In previous discussions of biological mortality bias in growth, some have argued that children in the past predominantly perished from acute illness, and that these illnesses could not have led to growth differences between survivors and non‐survivors (Cohen, 1994; Holman et al., 2004; Humphrey, 2000; Lovejoy et al., 1990; Sundick, 1978). We would disagree with this. As per the framework presented in *The Osteological Paradox* (Wood et al., 1992), selective mortality acts on hidden heterogeneity to produce the skeletal sample. Although a final cause of death may be acute illness, it is highly possible that a child experienced multiple bouts of illness through life that weakened them and made them more susceptible to death. Studies now show that children with less fat stores, that is, nutritionally compromised children, show substantial suppression of growth during illness relative to children with more fat stores (Urlacher et al., 2018). When this is added to the well‐known synergism between infection and malnutrition (Scrimshaw, 2003), it is easy to imagine that children who consistently experienced bouts of poor health could become simultaneously growth compromised and susceptible to death, producing biological mortality bias in growth. This is supported by observations from pre‐epidemiological transition societies wherein malnourished children suffered from higher mortality due to disease compared with better nourished peers (Birn et al., 2010; Díaz‐Briguets, 1981). Therefore, even if child deaths in the past were caused by acute illnesses, this does not preclude the possibility that biological mortality bias in growth affects skeletal samples from these populations.

Another argument used to minimize the potential impact of biological mortality bias is that because bioarchaeologists compare non‐survivors across populations, biological mortality bias is not a problem for paleoauxology. Others take a milder stance, arguing that it is minimally problematic as long as analyses rely on comparing growth between past populations and not to modern populations. While we would agree that the problem can in some cases be minimized, we disagree that is it always removed. It is important to remember that that selective mortality acts on hidden heterogeneity in order to produce non‐survivors or skeletal samples (Wood et al., 1992). By this framework, the presence and magnitude of hidden heterogeneity influences the potential for and magnitude of biological mortality bias. In thinking about health inequity and hidden heterogeneity, Frenk et al.' (1991) model is helpful. It posits five levels at which health inequities can be introduced: individual, household, institutional, societal, and systemic. Societal‐level inequality is relatively easy to see archeologically and gives information on the level of heterogeneity that may exist for selective mortality to act upon. Some societies, for example small, egalitarian, mobile foragers, are likely to have smaller variation in hidden heterogeneity than larger, differentiated, hierarchical, urbanized groups. The magnitude of biological mortality bias present in each population is modulated by its social and economic structure. As such, the extent to which the skeletal sample is affected by biological mortality bias can vary across populations, as others have pointed out (Eisenberg, 1992; Jankaukas & Česnys, 1992). Therefore, the impact of mortality bias is dependent on the type of societies being compared.

Some could argue that a modern sample, particularly from populations with access to medical care and steady sources of nutrition, may not yield estimates of biological mortality bias in growth that accurately reflects its impact on past populations. We would agree with this statement. However, there are very few options for studying biological mortality bias in growth other than modern samples, and this is likely why the topic has remained relatively untested since the publication of The Osteological Paradox (Wood et al., 1992). In order to quantify biological mortality bias in growth, two samples are needed: (1) a group of non‐surviving children, and (2) a group of surviving children from the same cohort who can be skeletally assessed at the same ages as the non‐survivors. In archaeological samples, survivors enter the archaeological records as adults. Identified collections such as the Lisbon sample (Cardoso, 2006) would be ideal if radiographs of children were available for the same period. Unfortunately, the only samples currently available to study biological mortality bias in growth consist of medical images from contemporary populations. We have attempted to address the discrepancy between modern and past populations by comparing our results to 20th‐century studies of anthropometrics in surviving and non‐surviving children in preindustrial groups (Table 8). Doing so suggests that the results of this study underestimate the magnitude of biological mortality bias in past populations.

Several studies have now attempted to examine mortality bias in diaphyseal growth: Saunders and Hoppa (1993), Spake and Cardoso (2019), Spake et al. (2020), Stull et al. (2021), and the present study. Overwhelmingly, these studies conclude that there is some mortality bias in diaphyseal growth, although there is no clear consensus on how influential it is on the results of bioarchaeological studies (Saunders & Hoppa, 1993, Spake & Cardoso, 2019, Spake, 2020, and this study). Stull et al. (2021), concluded that mortality bias was not present throughout ontogeny, although they did find that children dying of natural deaths under the age of 2 years were indeed smaller than children dying of other manners of death. The contrast between their study and ours is especially interesting as the study sampled partially overlap: both studies draw a portion of their samples from the OMI. The contrasting findings between our study and theirs could be due to statistical or sample selection procedures. The authors' analysis tested for differences in long bone lengths within each 1‐year increment. Some of the lack of differences between the manner of death groups may be due to sampling issues. For example, there were only a total of 10 individuals in the 10‐year age group for the US sample, which must then be separated across three manners of death—age distributions were not given for the manners of death separately. It is clear that at least with the South African samples, manner of death groups were not equally well‐represented across the age range: there were very few natural deaths older than 2 years of age, while accidental deaths were represented through the age range (Figure 3). These sampling issues are especially important given Stull and coworker's statistical choices: they tested for differences in the distribution of long bone lengths across manner of death groups within each 1 year increment without standardizing long bone length for age, meaning that these tests are sensitive to both small sample sizes as well as differences in age distribution within each year increment. In fact, examination of Stull et al.'s plot for the US (Figure 6) show that accident victims have the largest long bone length for age throughout the entire age range studies, not just for individuals under 2 years of age. Unfortunately, test statistics for the comparisons between manners of deaths were not provided. Lastly, Stull et al. make no attempt to consider how the results from their study of contemporary populations may extrapolate onto past populations who experienced vastly different socioecologies from contemporary children. These differences between our approaches may explain differences in results between this study and Stull and coworkers'.

Of the studies discussed here, Saunders and Hoppa's analysis, which simulated biological mortality bias in growth based on studies of anthropometrics and survivorship in nonindustrialized populations, comes the closest to studying biological mortality bias in growth in natural fertility and mortality populations, and therefore is the most likely to approximate what would be observed in archaeological populations. Based on the findings of all available studies on biological mortality bias in growth, the balance of evidence suggests that biological mortality bias in growth exist, although whether it poses an important problem for paleoauxologists, and if so, how to address it, remain questions that paleoauxologists must discuss as a discipline.

## 5 CONCLUSION

In this study, we document differences in growth status between survivors and non‐survivor. We found evidence for mortality bias in individuals over the age of 3 years, and potentially also in very young infants under the age of 6 months. This bias was more pronounced in females compared with males. We have estimated that differences in growth between survivors and non‐survivors is up to 2 *z*‐score units, which represents as much as 18 months' worth of growth in our sample. We have also shown that our sample underestimates the magnitude of biological mortality bias in growth in past populations, where it may be 1.5 to 5 times the magnitude found in our contemporary sample. We would consider a mortality bias effect of 2 *z*‐score units to be large: for comparison, a child whose height is 2 *z*‐score units below the mean in contemporary medical settings is considered stunted and at higher risk for poor cognitive performance, bouts of illness, and death (World Health Organization, 2010). Whether paleoauxologists agree with us that mortality bias in growth influences the results of bioarchaeological growth studies, we demonstrate that the potential for its impact exists.

This study identifies a clear and hereto unrecognized difference in the effect between males and females, wherein females are more susceptible to biological mortality bias than males. Previously, differences in skeletal growth between females and males have not been assessed in bioarchaeological research because of unreliable sex estimates due to limited sexual dimorphism prior to adolescence. This study emphasizes the importance of estimating sex in juveniles in past populations, which is now possible at reasonable cost using amelogenin peptide analysis (Stewart et al., 2016, 2017). Tests of this method on developing dentition has now shown that it can be used to consistently and reliably estimate sex in juveniles as young as perinate (Gowland et al., 2021; Stewart et al., 2017). As this method grows in popularity, we urge bioarchaeologists studying growth to make use of it to explore sex‐based differences in growth attainment within their study populations.

We suggest that biological mortality bias in growth remains an unresolved problem and must be further investigated in order to strengthen bioarchaeological research regarding the health status of past populations. Various advances in methods now allow paleoauxologists to better contextualize their samples. These include sex estimation with amelogenin peptide analysis, better age estimation methods including histological and nondestructive methods (Le Cabec et al., 2017; McFarlane et al., 2014), and serial stable isotope analysis to understand stress over the life course (e.g., McCool et al., 2021). Further, we suggest that in order to fully document the effect of biological mortality bias in growth, studies should consider how population variation in social and economic structure modulates the magnitude of biological mortality bias as described above. Whether biological mortality bias in growth is likely to be an influence on conclusions drawn from paleoauxological studies is dependent on the age and sex composition of the sample as well as the context from which it is drawn. Bioarchaeologists should carefully consider these factors in deciding whether biological mortality bias is likely to affect their results.

## CONFLICT OF INTEREST

The authors have no conflicts of interest to disclose.

## AUTHOR CONTRIBUTIONS

**Laure Spake:** Conceptualization (lead); data curation (lead); formal analysis (lead); funding acquisition (supporting); investigation (lead); methodology (lead); project administration (lead); writing – original draft (lead); writing – review and editing (lead). **Robert D. Hoppa:** Conceptualization (supporting); methodology (supporting); supervision (equal); writing – review and editing (supporting). **Soren Blau:** Resources (lead); software (supporting); supervision (supporting); writing – review and editing (supporting). **Hugo F. V. Cardoso:** Conceptualization (equal); funding acquisition (lead); methodology (equal); project administration (supporting); resources (supporting); supervision (lead); writing – review and editing (supporting).

## Supporting information


**Table S1**. Number of females (F) and males (M) aged in each 1 year increment of the age range, by manner of death, for the US (OMI), Australian (VIFM), and total sample (OMI and VIFM combined).
**Table S2**. Number of individuals (N) and mean and standard deviation (SD) for humerus length z‐scores for each of the manner of deaths. The t and p values for t‐tests between them and Cohen's d (d) for effect size are given. Values are calculated for the age groups separately and for the total sample.
**Table S3**. Number of individuals (N) and mean and standard deviation (SD) for radius length z‐scores for each of the manner of deaths. The t and p values for t‐tests between them and Cohen's d (d) for effect size are given. Values are calculated for the age groups separately and for the total sample.
**Table S4**. Number of individuals (N) and mean and standard deviation (SD) for ulna length z‐scores for each of the manner of deaths. The t and p values for t‐tests between them and Cohen's d (d) for effect size are given. Values are calculated for the age groups separately and for the total sample.
**Table S5**. Number of individuals (N) and mean and standard deviation (SD) for tibia length z‐scores for each of the manner of deaths. The t and p values for t‐tests between them and Cohen's d (d) for effect size are given. Values are calculated for the age groups separately and for the total sample.
**Table S6**. Number of individuals (N) and mean and standard deviation (SD) for fibula length z‐scores for each of the manner of deaths. The t and p values for t‐tests between them and Cohen's d (d) for effect size are given. Values are calculated for the age groups separately and for the total sample.
**Table S7**. Test statistic, p‐value for t‐tests and Mann–Whitney tests, and corresponding adjusted p‐value for differences in the distribution of various long bone length for age z‐scores between female survivors and non‐survivors in the child age group. p‐values were adjusted using the False Discovery Rate (FDR) method.

## Data Availability

Data are not publicly available due to privacy or ethical restrictions.
