# Supplementary material for: Biological mortality bias in diaphyseal growth of contemporary children: Implications for paleoauxology
Source: Am J Biol Anthropol. 2022 Jan 29;178(1):89–107. doi: 10.1002/ajpa.24486 (PMC9306609; doi:10.1002/ajpa.24486)
Supplement: Supplementary file 1 — Table S1. Number of females (F) and males (M) aged in each 1 year increment of the age range, by manner of death, for the US (OMI), Australian (VIFM), and total sample (OMI and VIFM combined). Table S2. Number of individuals (N) and mean and standard deviation (SD) for humerus length z‐scores for each of the manner of deaths. The t and p values for t‐tests between them and Cohen's d (d) for effect size are given. Values are calculated for the age groups separately and for the total sample. Table S3. Number of individuals (N) and mean and standard deviation (SD) for radius length z‐scores for each of the manner of deaths. The t and p values for t‐tests between them and Cohen's d (d) for effect size are given. Values are calculated for the age groups separately and for the total sample. Table S4. Number of individuals (N) and mean and standard deviation (SD) for ulna length z‐scores for each of the manner of deaths. The t and p values for t‐tests between them and Cohen's d (d) for effect size are given. Values are calculated for the age groups separately and for the total sample. Table S5. Number of individuals (N) and mean and standard deviation (SD) for tibia length z‐scores for each of the manner of deaths. The t and p values for t‐tests between them and Cohen's d (d) for effect size are given. Values are calculated for the age groups separately and for the total sample. Table S6. Number of individuals (N) and mean and standard deviation (SD) for fibula length z‐scores for each of the manner of deaths. The t and p values for t‐tests between them and Cohen's d (d) for effect size are given. Values are calculated for the age groups separately and for the total sample. Table S7. Test statistic, p‐value for t‐tests and Mann–Whitney tests, and corresponding adjusted p‐value for differences in the distribution of various long bone length for age z‐scores between female survivors and non‐survivors in the child age group. p‐values were adjusted using the False Discovery Ra [file AJPA-178-89-s001.docx]

**Supplementary Materials**

Table S1. Number of females (F) and males (M) aged in each 1 year increment of the age range, by manner of death, for the US (OMI), Australian (VIFM), and total sample (OMI and VIFM combined).

|  | **Age increment** | | | | | | | | | | | | |
| --- | --- | --- | --- | --- | --- | --- | --- | --- | --- | --- | --- | --- | --- |
|  | 0-0.99 | 1-  1.99 | 2-  2.99 | 3-  3.99 | 4-  4.99 | 5-  5.99 | 6-  6.99 | 7-  7.99 | 8-  8.99 | 9-  9.99 | 10-  10.99 | 11-  11.99 | 12-12.99 |
| **OMI** |  |  |  |  |  |  |  |  |  |  |  |  |  |
| **Accident** |  |  |  |  |  |  |  |  |  |  |  |  |  |
| Female | 2 | 5 | 4 | 1 | 3 | 3 | 2 | 4 | 1 | 2 | 0 | 1 | 2 |
| Male | 5 | 7 | 6 | 6 | 3 | 2 | 1 | 2 | 3 | 1 | 2 | 1 | 1 |
|  |  |  |  |  |  |  |  |  |  |  |  |  |  |
| **Natural** |  |  |  |  |  |  |  |  |  |  |  |  |  |
| Female | 0 | 4 | 1 | 2 | 1 | 0 | 0 | 1 | 0 | 0 | 0 | 0 | 0 |
| Male | 2 | 7 | 0 | 1 | 1 | 0 | 0 | 0 | 1 | 2 | 0 | 1 | 0 |
|  |  |  |  |  |  |  |  |  |  |  |  |  |  |
| **VIFM** |  |  |  |  |  |  |  |  |  |  |  |  |  |
| **Accident** |  |  |  |  |  |  |  |  |  |  |  |  |  |
| Female | 2 | 4 | 3 | 2 | 3 | 1 | 2 | 2 | 2 | 2 | 0 | 3 | 1 |
| Male | 2 | 2 | 3 | 2 | 2 | 4 | 2 | 3 | 3 | 3 | 2 | 3 | 3 |
|  |  |  |  |  |  |  |  |  |  |  |  |  |  |
| **Natural** |  |  |  |  |  |  |  |  |  |  |  |  |  |
| Female | 3 | 3 | 3 | 0 | 2 | 2 | 1 | 1 | 4 | 3 | 1 | 0 | 2 |
| Male | 4 | 2 | 2 | 3 | 1 | 1 | 1 | 2 | 0 | 0 | 3 | 1 | 1 |
|  |  |  |  |  |  |  |  |  |  |  |  |  |  |
| **Total** |  |  |  |  |  |  |  |  |  |  |  |  |  |
| **Accident** |  |  |  |  |  |  |  |  |  |  |  |  |  |
| Female | 4 | 9 | 7 | 3 | 6 | 4 | 4 | 6 | 3 | 4 | 0 | 4 | 3 |
| Male | 7 | 9 | 9 | 8 | 5 | 6 | 3 | 5 | 6 | 4 | 4 | 4 | 4 |
|  |  |  |  |  |  |  |  |  |  |  |  |  |  |
| **Natural** |  |  |  |  |  |  |  |  |  |  |  |  |  |
| Female | 3 | 7 | 4 | 2 | 3 | 2 | 1 | 2 | 4 | 3 | 1 | 0 | 2 |
| Male | 6 | 9 | 2 | 4 | 2 | 1 | 1 | 2 | 1 | 2 | 3 | 2 | 1 |
|  |  |  |  |  |  |  |  |  |  |  |  |  |  |

Table S2. Number of individuals (N) and mean and standard deviation (SD) for humerus length z-scores for each of the manner of deaths. The t and p values for t-tests between them and Cohen’s d (d) for effect size are given. Values are calculated for the age groups separately and for the total sample.

|  | Accident | | | Natural | | |  |  |  |
| --- | --- | --- | --- | --- | --- | --- | --- | --- | --- |
|  | N | Mean | SD | N | Mean | SD | t | p | d |
| **Sexes combined** |  |  |  |  |  |  |  |  |  |
| Infant | 45 | 1.05 | 1.24 | 30 | 0.99 | 0.90 | 0.24 | 0.81 | 0.05 |
| Child | 38 | 0.93 | 1.32 | 16 | 0.27 | 1.25 | 1.73 | 0.09 | 0.51 |
| Juvenile | 33 | 0.63 | 1.40 | 19 | 0.26 | 1.71 | 0.81 | 0.42 | 0.25 |
| Total | 116 | 0.89 | 1.31 | 65 | 0.60 | 1.30 | 1.45 | 0.15 | 0.22 |
| **Females** |  |  |  |  |  |  |  |  |  |
| Infant | 20 | 1.04 | 1.38 | 13 | 1.12 | 1.19 | -0.18 | 0.86 | 0.06 |
| Child | 16 | 1.23 | 1.36 | 8 | -0.42 | 1.12 | 3.15 | 0.01 | 1.28 |
| Juvenile | 13 | 0.51 | 1.54 | 9 | -0.33 | 1.01 | 1.53 | 0.14 | 0.62 |
| Total | 49 | 0.96 | 1.42 | 30 | 0.28 | 1.32 | 2.17 | 0.03 | 0.49 |
| **Males** |  |  |  |  |  |  |  |  |  |
| Infant | 25 | 1.07 | 1.13 | 17 | 0.90 | 0.60 | 0.63 | 0.53 | 0.18 |
| Child | 22 | 0.71 | 1.28 | 8 | 0.96 | 1.00 | -0.50 | 0.62 | 0.19 |
| Juvenile | 20 | 0.71 | 1.34 | 10 | 0.78 | 2.07 | -0.09 | 0.92 | 0.04 |
| Total | 67 | 0.84 | 1.24 | 0.88 | 1.23 | 1.40 | -0.13 | 0.89 | 0.03 |

Table S3. Number of individuals (N) and mean and standard deviation (SD) for radius length z-scores for each of the manner of deaths. The t and p values for t-tests between them and Cohen’s d (d) for effect size are given. Values are calculated for the age groups separately and for the total sample.

|  | Accident | | | Natural | | |  |  |  |
| --- | --- | --- | --- | --- | --- | --- | --- | --- | --- |
|  | N | Mean | SD | N | Mean | SD | t | p | d |
| **Sexes combined** |  |  |  |  |  |  |  |  |  |
| Infant | 44 | 0.86 | 1.22 | 30 | 1.00 | 1.13 | -0.53 | 0.60 | 0.12 |
| Child | 37 | 0.93 | 1.12 | 15 | 0.37 | 1.40 | 1.37 | 0.18 | 0.46 |
| Juvenile | 39 | 0.68 | 1.39 | 20 | 0.50 | 1.65 | 0.42 | 0.68 | 0.12 |
| Total | 120 | 0.82 | 1.24 | 65 | 0.70 | 1.38 | 0.58 | 0.57 | 0.09 |
| **Females** |  |  |  |  |  |  |  |  |  |
| Infant | 20 | 0.94 | 1.31 | 13 | 1.40 | 1.47 | -0.88 | 0.39 | 0.32 |
| Child | 15 | 1.21 | 1.09 | 7 | -0.43 | 1.16 | 3.13 | 0.01 | 1.47 |
| Juvenile | 16 | 0.30 | 1.29 | 10 | -0.15 | 0.72 | 1.14 | 0.27 | 0.40 |
| Total | 51 | 0.82 | 1.28 | 30 | 0.45 | 1.43 | 1.16 | 0.25 | 0.28 |
| **Males** |  |  |  |  |  |  |  |  |  |
| Infant | 24 | 0.78 | 1.16 | 17 | 0.71 | 0.67 | 0.26 | 0.79 | 0.08 |
| Child | 22 | 0.73 | 1.12 | 8 | 1.07 | 1.25 | -0.66 | 0.52 | -0.29 |
| Juvenile | 23 | 0.94 | 1.42 | 10 | 1.15 | 2.07 | -0.28 | 0.78 | 0.12 |
| Total | 69 | 0.82 | 1.23 | 35 | 0.92 | 1.31 | -0.35 | 0.72 | 0.08 |

Table S4. Number of individuals (N) and mean and standard deviation (SD) for ulna length z-scores for each of the manner of deaths. The t and p values for t-tests between them and Cohen’s d (d) for effect size are given. Values are calculated for the age groups separately and for the total sample.

|  | Accident | | | Natural | | |  |  |  |
| --- | --- | --- | --- | --- | --- | --- | --- | --- | --- |
|  | N | Mean | SD | N | Mean | SD | t | p | d |
| **Sexes combined** |  |  |  |  |  |  |  |  |  |
| Infant | 44 | 0.96 | 1.19 | 30 | 1.06 | 1.15 | -0.37 | 0.71 | 0.09 |
| Child | 37 | 1.19 | 1.20 | 14 | 0.48 | 1.68 | 1.44 | 0.17 | 0.53 |
| Juvenile | 38 | 0.77 | 1.52 | 20 | 0.51 | 1.72 | 0.57 | 0.57 | 0.16 |
| Total | 119 | 0.97 | 1.31 | 64 | 0.76 | 1.47 | 0.95 | 0.35 | 0.15 |
| **Females** |  |  |  |  |  |  |  |  |  |
| Infant | 20 | 1.07 | 1.26 | 13 | 1.32 | 1.50 | -0.49 | 0.63 | 0.18 |
| Child | 15 | 1.38 | 1.15 | 7 | -0.42 | 1.47 | 2.86 | 0.02 | 1.44 |
| Juvenile | 16 | 0.42 | 1.55 | 10 | -0.14 | 0.90 | 1.16 | 0.26 | 0.42 |
| Total | 51 | 0.96 | 1.36 | 30 | 0.43 | 1.50 | 1.59 | 0.11 | 0.38 |
| **Males** |  |  |  |  |  |  |  |  |  |
| Infant | 24 | 0.87 | 1.14 | 17 | 0.87 | 0.78 | 0.01 | 0.99 | <0.01 |
| Child | 22 | 1.05 | 1.25 | 7 | 1.38 | 1.44 | -0.54 | 0.60 | 0.25 |
| Juvenile | 22 | 1.03 | 1.47 | 10 | 1.16 | 2.12 | -0.18 | 0.86 | 0.08 |
| Total | 68 | 0.98 | 1.27 | 34 | 1.06 | 1.39 | -0.28 | 0.78 | 0.06 |

Table S5. Number of individuals (N) and mean and standard deviation (SD) for tibia length z-scores for each of the manner of deaths. The t and p values for t-tests between them and Cohen’s d (d) for effect size are given. Values are calculated for the age groups separately and for the total sample.

|  | Accident | | | Natural | | |  |  |  |
| --- | --- | --- | --- | --- | --- | --- | --- | --- | --- |
|  | N | Mean | SD | N | Mean | SD | t | p | d |
| **Sexes combined** |  |  |  |  |  |  |  |  |  |
| Infant | 44 | 1.09 | 1.22 | 30 | 1.11 | 1.23 | -0.09 | 0.93 | 0.02 |
| Child | 37 | 0.62 | 1.26 | 16 | 0.06 | 1.45 | 1.35 | 0.19 | 0.43 |
| Juvenile | 40 | 0.33 | 1.27 | 20 | 0.01 | 1.55 | 0.77 | 0.45 | 0.23 |
| Total | 121 | 0.69 | 1.28 | 66 | 0.53 | 1.47 | 0.78 | 0.44 | 0.12 |
| **Females** |  |  |  |  |  |  |  |  |  |
| Infant | 19 | 1.12 | 1.40 | 13 | 1.54 | 1.38 | -0.85 | 0.40 | 0.31 |
| Child | 16 | 1.03 | 1.27 | 8 | -0.63 | 1.44 | 2.77 | 0.02 | 1.25 |
| Juvenile | 17 | 0.07 | 1.29 | 10 | -0.47 | 1.09 | 1.15 | 0.26 | 0.44 |
| Total | 52 | 0.75 | 1.39 | 31 | 0.33 | 1.64 | 1.18 | 0.25 | 0.28 |
| **Males** |  |  |  |  |  |  |  |  |  |
| Infant | 25 | 1.06 | 1.09 | 17 | 0.78 | 1.02 | 0.85 | 0.40 | 0.26 |
| Child | 21 | 0.31 | 1.18 | 8 | 0.75 | 1.16 | -0.90 | 0.38 | 0.37 |
| Juvenile | 23 | 0.51 | 1.24 | 10 | 0.50 | 1.84 | 0.03 | 0.98 | 0.01 |
| Total | 69 | 0.65 | 1.20 | 35 | 0.69 | 1.30 | -0.16 | 0.87 | 0.03 |

Table S6. Number of individuals (N) and mean and standard deviation (SD) for fibula length z-scores for each of the manner of deaths. The t and p values for t-tests between them and Cohen’s d (d) for effect size are given. Values are calculated for the age groups separately and for the total sample.

|  | Accident | | | Natural | | |  |  |  |
| --- | --- | --- | --- | --- | --- | --- | --- | --- | --- |
|  | N | Mean | SD | N | Mean | SD | t | p | d |
| **Sexes combined** |  |  |  |  |  |  |  |  |  |
| Infant | 44 | 1.02 | 1.26 | 30 | 1.09 | 1.34 | -0.24 | 0.81 | 0.06 |
| Child | 37 | 0.71 | 1.31 | 16 | -0.07 | 1.49 | 1.81 | 0.08 | 0.57 |
| Juvenile | 39 | 0.32 | 1.24 | 20 | 0.07 | 1.41 | 0.70 | 0.49 | 0.20 |
| Total | 120 | 0.70 | 1.29 | 66 | 0.50 | 1.48 | 0.92 | 0.36 | 0.15 |
| **Females** |  |  |  |  |  |  |  |  |  |
| Infant | 19 | 1.05 | 1.48 | 13 | 1.46 | 1.57 | -0.76 | 0.46 | 0.28 |
| Child | 16 | 1.10 | 1.29 | 8 | -0.83 | 1.40 | 3.25 | 0.01 | 1.48 |
| Juvenile | 16 | 0.17 | 1.31 | 10 | -0.23 | 0.75 | 1.00 | 0.33 | 0.36 |
| Total | 51 | 0.79 | 1.41 | 31 | 0.33 | 1.62 | 1.31 | 0.19 | 0.31 |
| **Males** |  |  |  |  |  |  |  |  |  |
| Infant | 25 | 0.99 | 1.10 | 17 | 0.80 | 1.09 | 0.55 | 0.59 | 0.17 |
| Child | 21 | 0.41 | 1.27 | 8 | 0.68 | 1.23 | -0.52 | 0.61 | 0.21 |
| Juvenile | 23 | 0.43 | 1.20 | 10 | 0.36 | 1.86 | 0.11 | 0.92 | 0.05 |
| Total | 69 | 0.63 | 1.20 | 35 | 0.65 | 1.35 | -0.08 | 0.94 | 0.02 |

Table S7. Test statistic, p-value for t-tests and Mann-Whitney tests, and corresponding adjusted p-value for differences in the distribution of various long bone length for age z-scores between female survivors and non-survivors in the child age group. p-values were adjusted using the False Discovery Rate (FDR) method.

|  | **Welch’s t** | | | **Mann Whitney U** | | |
| --- | --- | --- | --- | --- | --- | --- |
|  | Test statistic | p-value | Adjusted p-value | Test statistic | p-value | Adjusted p-value |
| **Females, child age group** |  |  |  |  |  |  |
| Humerus | 3.14 | 0.006 | 0.015 | 105 | 0.011 | 0.013 |
| Radius | 3.13 | 0.009 | 0.015 | 92 | 0.004 | 0.013 |
| Ulna | 2.86 | 0.018 | 0.018 | 87 | 0.014 | 0.014 |
| Femur | 2.96 | 0.010 | 0.015 | 94 | 0.010 | 0.013 |
| Tibia | 2.77 | 0.016 | 0.018 | 106 | 0.009 | 0.013 |
| Fibula | 3.25 | 0.006 | 0.015 | 109 | 0.005 | 0.013 |
